# Supplementary material for: Comparative genomics provides new insights into the diversity, physiology, and sexuality of the only industrially exploited tremellomycete: Phaffia rhodozyma
Source: BMC Genomics. 2016 Nov 9;17:901. doi: 10.1186/s12864-016-3244-7 (PMC5103461; doi:10.1186/s12864-016-3244-7)
Supplement: Additional file 6: — List of orphan genes with links to PFAM (related to Additional file 1: Table S1). (ZIP 1428 kb) [file 12864_2016_3244_MOESM6_ESM.zip › PFAM_UNIQUE/G01771_P.html]

#                                                                            --- full sequence --- -------------- this domain -------------   hmm coord   ali coord   env coord
  
# target name        accession   tlen query name           accession   qlen   E-value  score  bias   #  of  c-Evalue  i-Evalue  score  bias  from    to  from    to  from    to  acc description of target
  
#------------------- ---------- ----- -------------------- ---------- ----- --------- ------ ----- --- --- --------- --------- ------ ----- ----- ----- ----- ----- ----- ----- ---- ---------------------
  
G01771\_P             -            284 Baculo\_PEP\_C         PF04513.7    140     0.041   12.4  22.9   1   3     0.016       4.1    5.9   3.1    88   113    46    71    37    81 0.73 - Baculovirus polyhedron envelope protein, PEP, C terminus  
G01771\_P             -            284 Baculo\_PEP\_C         PF04513.7    140     0.041   12.4  22.9   2   3     0.066        17    3.9   1.6    87   139    78   135    73   137 0.66 - Baculovirus polyhedron envelope protein, PEP, C terminus  
G01771\_P             -            284 Baculo\_PEP\_C         PF04513.7    140     0.041   12.4  22.9   3   3   0.00026     0.069   11.7   4.9    34   106   174   244   164   271 0.66 - Baculovirus polyhedron envelope protein, PEP, C terminus  
G01771\_P             -            284 HrcA                 PF01628.16   224     0.061   11.6   0.1   1   2      0.28   8.5e+02   -2.0   0.0     8    23    52    67    48    76 0.82 - HrcA protein C terminal domain  
G01771\_P             -            284 HrcA                 PF01628.16   224     0.061   11.6   0.1   2   2   5.5e-05      0.16   10.2   0.1    52   121    70   139    55   153 0.88 - HrcA protein C terminal domain
